# Absorption of silicon from artesian aquifer water and its impact on bone health in postmenopausal women: a 12 week pilot study

**DOI:** 10.1186/1475-2891-9-44

**Published:** 2010-10-14

**Authors:** Zhaoping Li, Hannah Karp, Alona Zerlin, Tsz Ying Amy Lee, Catherine Carpenter, David Heber

**Affiliations:** 1 Center for Human Nutrition, University of California Los Angeles, Los Angeles, CA, USA

## Abstract

**Background:**

Decreased bone mineral density and osteoporosis in postmenopausal women represents a growing source of physical limitations and financial concerns in our aging population. While appropriate medical treatments such as bisphosphonate drugs and hormone replacement therapy exist, they are associated with serious side effects such as osteonecrosis of the jaw or increased cardiovascular risk. In addition to calcium and vitamin D supplementation, previous studies have demonstrated a beneficial effect of dietary silicon on bone health. This study evaluated the absorption of silicon from bottled artesian aquifer water and its effect on markers of bone metabolism.

**Methods:**

Seventeen postmenopausal women with low bone mass, but without osteopenia or osteoporosis as determined by dual x-ray absorptiometry (DEXA) were randomized to drink one liter daily of either purified water of low-silicon content (PW) or silicon-rich artesian aquifer water (SW) (86 mg/L silica) for 12 weeks. Urinary silicon and serum markers of bone metabolism were measured at baseline and after 12 weeks and analyzed with two-sided t-tests with p < 0.05 defined as significant.

**Results:**

The urinary silicon level increased significantly from 0.016 ± 0.010 mg/mg creatinine at baseline to 0.037 ± 0.014 mg/mg creatinine at week 12 in the SW group (p = 0.003), but there was no change for the PW group (0.010 ± 0.004 mg/mg creatinine at baseline vs. 0.009 ± 0.006 mg/mg creatinine at week 12, p = 0.679). The urinary silicon for the SW group was significantly higher in the silicon-rich water group compared to the purified water group (p < 0.01). NTx, a urinary marker of bone resorption did not change during the study and was not affected by the silicon water supplementation. No significant change was observed in the serum markers of bone formation compared to baseline measurements for either group.

**Conclusions:**

These findings indicate that bottled water from artesian aquifers is a safe and effective way of providing easily absorbed dietary silicon to the body. Although the silicon did not affect bone turnover markers in the short-term, the mineral's potential as an alternative prevention or treatment to drug therapy for osteoporosis warrants further longer-term investigation in the future.

**Trial Registration:**

ClinicalTrials.gov Identifier: NCT01067508

## Background

Osteoporosis is a leading cause of morbidity and mortality in the elderly [1]. In the U.S., an estimated 4-6 million women aged >50 years have osteoporosis, and another 13-17 million (37%-50%) have osteopenia (or low bone density) based on femoral bone mineral density (BMD) tests using dual x-ray absorptiometry (DEXA) when osteoporosis is defined by a T-score of less than -2 while osteopenia is defined as having a T-score between -2 and -1.5 [1]. As a result of demographic shifts and an aging population in the U.S. there has been a significant increase in the number of adults with low femoral neck BMD (osteoporosis + osteopenia) in 2005-2006 compared to the number identified between 1988 and1994 [2]. Osteoporosis causes 1.5 million fractures in the U.S. annually, including 300,000 hip fractures and 700,000 vertebral spine fractures [3]. The estimated cost of treating osteoporotic fractures in 2005 was $17 billion, and this cost is expected to increase by 50% by 2025 as the population ages [4].

Beyond genetic and hormonal factors that affect bone density with aging, lifestyle factors including inadequate intake of calcium and vitamin D, smoking, excessive alcohol use, regular consumption of soft drinks, lack of physical exercise, and lack of fruit and vegetable intake exacerbate decreases in bone density which increase the risk for bone fracture [5-8]. Clinicians commonly recommend calcium and vitamin D supplementation, bisphosphonate drugs, strontium ranelate, calcitonin, parathyroid hormone, estrogen, and some anti-estrogens with weak estrogen effects to women with reduced bone density in order to reduce bone turnover, but have paid less attention to nutritional factors such as reducing soft drink consumption [9]. While appropriate for treating osteoporosis, these medical approaches have been associated with serious side effects such as osteonecrosis of the jaw in patients receiving oral bisphosphonates [10], and increased risks of breast cancer, stroke, and venous thromboembolism in women treated with postmenopausal hormone replacement therapy [11].

Silicon (Si) is the most abundant trace element in the diet after iron and zinc. In 1970, Carlisle suggested that silicon is a possible factor in bone calcification [12]. The dietary consumption of silicon and other trace minerals has been associated positively with bone mass, while mineral deficiencies have been associated with reduced bone density [13,14]. Animal studies in the 1970's reported that dietary silicon deficiency resulted in reduced bone tensile strength [15,16]. In the Framingham offspring cohort, increased dietary silicon intake was associated with increased bone mass [17].

Drinking water and other fluids provide a significant potential dietary source of silicon, since silicon is primarily present in water as Si(OH)_4 _[18]. Bottled water from artesian aquifers contains silicon obtained from volcanic rock while typical bottled water, an increasingly popular alternative to soft drinks, is purified through reverse osmosis and does not contain significant amounts of any trace minerals. In comparison with the silicon-rich water which contains 85 to 90 mg/L silica, the purified water contains undetectable amount of silica. Beer contains 0.9 to 3.94 mg silicon/100 gram and is another potentially beneficial source of silicon for healthy bone [19]. However, heavy beer drinking is associated with a reduced trabecular bone density in the spine [20]. Water originating from artesian aquifers represents a good source of dietary silicon, without the disadvantages arising from increased consumption of beer. The present study was carried out in postmenopausal women with reduced bone density confirmed by DEXA in order to determine the absorption of silicon from artesian drinking water and its effects on bone remodeling markers as assessed by accepted metabolic markers in addition to calcium and vitamin D supplementation..

## Methods

### Subjects

Postmenopausal women within 5 years of menopause were recruited into the study. They were required to have reduced bone density but no evidence of osteoporosis or osteopenia by DEXA scan (T ≥ -1.5 in the total hip and lumbar spine). Women with a body mass index (BMI) >30 kg/m^2 ^or who were being treated with estrogens, corticosteroids, or bisphosphonates, who consumed more than one alcoholic beverage a day, or who had significant illness affecting bone metabolism were excluded.

The study protocol was approved by the Institutional Review Board at the University of California, Los Angeles (UCLA) and was conducted at the UCLA Center for Human Nutrition. All subjects gave written informed consent prior to participation in the study. All study methods and procedures were conducted in accordance with the ethical standards of the Declaration of Helsinki and Good Clinical Practice Guidelines.

### Study Design

This was a 12 week, randomized, placebo-controlled, interventional study. Subjects who met the inclusion and exclusion criteria were instructed to take 1200 mg of calcium and 800 IU of vitamin D (Caltrate 600+D Calcium Supplement, Wyeth Consumer Healthcare, Madison, NJ, USA) daily during a two week run-in and to continue this supplementation throughout the study. At baseline, subjects were randomized either to the silicon-rich water group (SW) or the purified water group of low-silicon content (PW). The SW group received bottled FIJI Water^® ^(FIJI Water LLC, Los Angeles, CA, USA) and the PW group received purified bottled water (Aguafina^® ^water, Pepsico, Inc.). Subjects were instructed to drink one liter of study provided water daily directly from the bottle. New batch of water was dispensed and empty water bottles were collected every 4 weeks to ensure compliance with the protocol. Subjects were instructed not to alter their activity level or diets in any way outside of consuming the water and supplements provided to them by the study.

### Measurements

A basic clinical examination including measurement of body weight, systolic and diastolic blood pressure and heart rate was performed at screening, baseline visit and week 12. All subjects were instructed to fast overnight 10 hours prior to their clinic visits. Fasting blood samples (18 ml per time point) and fasting single void, midstream spot urine samples were collected at screening, baseline and week 12 during morning appointments. The blood samples were measured for 25-hydroxy vitamin D, parathyroid hormone (PTH), procollagen type I intact N-terminal propeptide, bone specific alkaline phosphatase and osteocalcin; urine samples were analyzed for silicon, creatinine, calcium and collagen type 1 cross-linked N-telopeptide (NTx).

Urine silicon concentration was measured by NMS Labs (Willow Grove, PA, USA) using inductively coupled plasma atomic emission spectroscopy (ICP-AES). The calibration range is from 1 to 10 mcg/L with two urine controls at 4 and 10 mg/L. These were prepared using a 10 mg/mL solution of silicone in water with 0.4% F-. The %CV for the low control was 6.4 and for the high control was 5.2. All the other blood and urine samples were analyzed by the Ronald Reagan UCLA Medical Center Clinical Laboratory (CLIA and CAP certified) using commercially available kits.

### Statistical analysis

Results are expressed as means ± standard deviation (SD). Differences between the two groups were evaluated with t-tests. All tests are two-sided and p < 0.05 was defined as significant.

## Results

### Bottled waters

The silica content in the bottled water was measured at MWH Laboratories (Monrovia, California, USA) by the method of EPA 200.7-ICP and the test results met all NELAC (National Environmental Laboratory Accreditation Conference) requirements. The silicon-rich water contained 86 mg/L of silica while the purified bottled water contained no detectable amount.

### Subjects

41 post-menopausal women with reduced bone density were screened for the study. Twenty-two subjects did not meet the inclusion criteria and were excluded from participation. Two subjects withdrew from the study after the baseline visit, one due to time constraints and the other at the request of the investigator concerning for her cardiovascular health. The remaining 17 women completed the study. Both PW and SW were well tolerated without adverse events.

The average age was 54 years, BMI of 24.1 kg/m^2 ^and a t-score of -0.45 in the hip and -0.3 in the spine by DEXA. Both groups were well matched in age, BMI and bone density (Table 1). There were more White women in the SW and more Asian women in PW groups.

**Table 1:** Baseline characteristics of study subjects

		Silicon-rich Water	Purified Water
Number of subject		10	9
			
Age (years)		53.1 ± 6.5	54.4 ± 3.9
			
BMI (kg/m^2^)		23.4 ± 3.3	24.8 ± 4.5
			
Ethnicity	White	7	3
	Hispanic	0	1
	Asian	0	3
	African American	2	2
			
T-score	Total Hip	-0.6 ± 1.1	-0.5 ± 1.5
	Lumbar Spine	-0.4 ± 1.6	-0.2 ± 1.2
			
BMD (g/cm^2^)	Total Hip	0.9 ± 0.1	1.0 ± 0.3
	Lumbar Spine	1.2 ± 0.2	1.2 ± 0.3

Values are listed as mean ± SD.

### Urinary silicon

**Figure 1:**
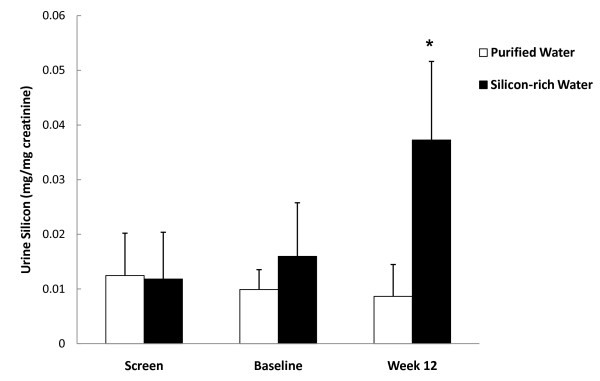
**Urine silicon**. Mean ± SD. Fasting spot urine was collected for urine creatinine and silicon. *silicon increased significantly (p < 0.05) in the silicon-rich water group from baseline to Week 12. No change was seen for the purified water group.

The urinary creatinine was compatible between the groups at baseline (82.2 ± 46.6 mg/dL in PW vs. 78.8 ± 43.2 mg/dL in SW groups) and there was not any significant change within groups during the study (p = 0.878). The screening urinary silicon was 0.012 ± 0.008 mg/mg creatinine in SW group and 0.012 ± 0.008 mg/mg creatinine in PW group, obtained from a fasting single spot collection. There was a decrease of silicon between screen and baseline for the PW group but was not statistically significant. The urinary silicon level increased significantly from 0.016 ± 0.010 mg/mg creatinine at baseline to 0.037 ± 0.014 mg/mg creatinine at week 12 in the SW group (p = 0.003), but there was no change for the PW group (0.010 ± 0.004 mg/mg creatinine at baseline vs. 0.009 ± 0.006 mg/mg creatinine at week 12, p = 0.679). At the end of the study, the urinary silicon for the SW group increased by 133.5% which was a statistically significant increase by comparison to the PW group (p < 0.01) (Figure 1).

### Urinary N-telopeptide

Urinary collagen type 1 cross-linked N-telopeptide excretion was normalized to creatinine in a fasting spot urine sample as a reflection of daily NTx. There was not any significant difference for baseline NTx levels between the two groups (36.0 ± 18.0 nmol/mmol in SW vs. 27.2 ± 11.5 nmol/mmol creatinine in PW). At the end of the study, differences between the groups were insignificant (36.0 ± 17.4 nmol/mmol in SW vs. 27.4 ± 8.1 nmol/mmol in PW, p = 0.250). There was not any statistic difference within groups either.

### Serum bone turnover markers

All the subjects had normal calcium levels during the entire study. One subject in the PW group had low vitamin D 25-hydroxy level (15 ng/ml) in spite of vitamin D supplementation. Baseline bone metabolism marker levels were not significantly different between groups. There as a wide variation in bone marker level in both groups at baseline and week 12. There was no change of parathyroid hormone or markers of bone formation including procollagen type I intact, N-terminal propeptide, bone specific alkaline phosphatase, and osteocalcin within groups or between groups (Table 2).

**Table 2:** Serum bone turnover markers

	Silicon-rich Water			Purified Water		
	
	Screen	Baseline	Week 12	Screen	Baseline	Week 12
Calcium (mg/L)	94.3 ± 1.4	93.7 ± 3.4	92.6 ± 3.4	94.7 ± 3.5	94.0 ± 3.9	93.3 ± 3.4
Bone specific Alk Phos (mcg/L)	13.9 ± 4.3	13.4 ± 4.1	13.0 ± 3.4	13.6 ± 13.4	14.5 ± 3.0	13.3 ± 3.4
Osteocalcin (ng/mL)	21.4 ± 8.0	18.0 ± 6.7	20.3 ± 6.8	20.7 ± 6.3	15.1 ± 4.0	17.7 ± 5.0
Procollagen t1 propeptide (μg/L)	62.3 ± 31.4	62.6 ± 28.3	55.8 ± 21.8	46.7 ± 22.1	57.6 ± 14.7	56.0 ± 19.1
PTH intact (pg/mL)	39.0 ± 9.6	33.3 ± 10.9	37.4 ± 12.2	38.4 ± 12.4	37.3 ± 15.4	44.1 ± 21.6
Vitamin D, 25 hydroxy (ng/mL)	29.2 ± 12.6	27.8 ± 10.4	27.0 ± 8.1	23.1 ± 8.2	23.5 ± 7.1	27.9 ± 11.1

Values are listed as mean ± SD.

## Discussion

Since the silicon deprivation studies [15,16] that suggested a potential role of silicon in bone and connective tissue health, there have been many studies investigating the potential role of dietary silicon in bone health [21]. Schiano et al reported that supplementation with soluble salt of silicium resulted in a significant increase in the total bone volume both in drinkable and in injectable form [22]. In a later study, silicon was shown to be more effective than Etidronate and sodium fluoride over a 14 to 22 month period [23]. In a recent double blind, placebo-controlled 12 month trial in osteopenic and osteoporotic subjects, Spector et al [24] reported that oral choline-stabilized orthosilicic acid had potential beneficial effects on bone collagen and a trend for a dose-related decrease in the bone resorption marker, collagen type 1 C-terminal telopeptide at 6 and 12 months.

The primary aim of the present study was to investigate the absorption of bottled silicon water. As much as 50% of ingested silicon is excreted in the urine [25]. Urinary excretion of silicon is a good marker of absorbed silicon and correlates well with dietary intake of silicon [19]. We clearly demonstrated that 86 mg/day of silica provided with bottled water significantly increased the urinary excretion of silicon.

The main route of entry of silicon to the body is from the gastrointestinal tract. Gastrointestinal uptake of silicon from foods was estimated in women as 24 mg/day and men as 30 mg per day. The major food sources are beer and bananas in men, and bananas and string beans in women [26]. It should be noted, however, that higher intakes of alcohol are associated with reduced bone density. Foods can be a major source of silicon [27,28], but in our study the intake of silicon-rich water from an artesian aquifer significantly increased silicon excretion over what was being excreted from dietary sources. Asians and Indians have much higher silicon intakes than do Western populations [29,30], and also have a lower incidence of hip fracture [31]. In addition, silicon intake decreases with age, with an average 0.1 mg less for every additional year of age [26]. Bottled silicon-rich water can be an additional source to improve silicon intake.

The present study did not demonstrate any significant change of bone metabolism markers in 12 weeks. All the study subjects were postmenopausal women. In the Framingham offspring cohort study Jugdaohsingh et al found that silicon intake was positively associated with bone mineral density for men and premenopausal women but not for postmenopausal women [17]. Men or premenopausal women may respond to silicon rich water differently. In the osteoporosis drug treatment studies the bone metabolism markers are mostly measured no earlier than 6 month [32-34]. This study was only 12 weeks. A longer supplementation period or lower BMD group may therefore have shown a greater effect of Si. It has been previously suggested that the effect of silicon is best under low calcium intake. Indeed a recent study by Kim et al [35] reported that Si supplementation increased bone mineral density in ovariectomised rats, only when the feed was deficient in calcium. No effect was seen under calcium replete or high calcium intake group. Finally change of short-term markers of bone resorption may not be the only mechanisms by which silicon improves bone health.

There have been conflicting reports on the influence of calcium and vitamin D on bone metabolic markers demonstrating no effect on markers of bone turnover but effects on bone loss [36,37]. To eliminate the potential effects of vitamin D and calcium deficiency on bone markers, all subjects in the present study were supplemented with vitamin D and calcium. We did not see any additional benefit of silicon supplementation on short-term markers of bone remodeling over 12 weeks.

Further research including studies over several years examining changes in bone density following long-term daily consumption of silicon-rich water obtained from artesian aquifers in women with reduced bone density are needed.

## Conclusions

This study clearly demonstrated that 86 mg/day of silica from bottled water increased urinary excretion of silicon over a 12-week period in post-menopausal women. This indicates that artesian aquifer bottled water is a safe and effective way of providing easily absorbed dietary silicon to the body. Although the silicon did not affect bone turnover in the short-term, the mineral's potential as an alternative prevention or treatment to drug therapy for osteoporosis warrants further, longer-term investigation in the future.

## Competing interests

The authors declare that they have no competing interests.

## Authors' contributions

ZL conceived of the study, participated in its design and coordination, performed statistical analysis, and draft the manuscript. HK coordinated the study and helped to draft the manuscript. AZ provided the dietary counsel for the study. TYL participated in subject screening and assessment. CC participated in subject assessment and assisted with statistical analysis. DH conceived of the study and helped draft the manuscript. All authors read and approved the final manuscript
